# Enhanced RAD21 cohesin expression confers poor prognosis and resistance to chemotherapy in high grade luminal, basal and HER2 breast cancers

**DOI:** 10.1186/bcr2814

**Published:** 2011-01-21

**Authors:** Huiling Xu, Max Yan, Jennifer Patra, Rachael Natrajan, Yuqian Yan, Sigrid Swagemakers, Jonathan M Tomaszewski, Sandra Verschoor, Ewan KA Millar, Peter van der Spek, Jorge S Reis-Filho, Robert G Ramsay, Sandra A O'Toole, Catriona M McNeil, Robert L Sutherland, Michael J McKay, Stephen B Fox

**Affiliations:** 1 Research Division, Peter MacCallum Cancer Centre, Locked Bag 1, A'Beckett Street, Melbourne, Vic 8006, Australia; 2 Department of Pathology, Peter MacCallum Cancer Centre, Locked Bag 1, A'Beckett Street, Melbourne, Vic 8006, Australia; 3 Department of Pathology, Faculty of Medicine and Dental Sciences, The University of Melbourne, Elizabeth Street, Parkville, Vic 3000, Australia; 4 The Breakthrough Breast Cancer Research Centre, Institute of Cancer Research, 237 Fulhan Road, London SW3 6JB, UK; 5 Department of Bioinformatics, Erasmus University Medical Centre, Dr. Molewaterplein 50, 3015 GE Rotterdam, The Netherlands; 6 Department of Genetics, Erasmus University Medical Centre, and Cancer Genomics Centre, Dr. Molewaterplein 50, 3015 GE Rotterdam, The Netherlands; 7 Department of Radiation Oncology, Peter MacCallum Cancer Centre, Locked Bag 1, A'Beckett Street, Melbourne, Vic 8006, Australia; 8 Cancer Research Program, Garvan Institute of Medical Research, 384 Victoria Street, Darlinghurst, NSW 2010, Australia; 9 Department of Anatomical Pathology, South Eastern Area Laboratory Service, St George Hospital, Gary Street, Kogarah, NSW 2217, Australia; 10 School of Medical Sciences, University of NSW, High Street, Kensington, NSW 2052, Australia; 11 School of Medicine, University of Western Sydney, Locked Bag 1797, Penrith, NSW 2751, Australia; 12 St Vincent's Clinical School, University of NSW, Victoria Street, Darlinghurst, NSW 2010, Australia; 13 Department of Tissue Pathology and Diagnostic Oncology, Royal Prince Alfred Hospital, Missenden Road, Camperdown, NSW 2050, Australia; 14 Central Clinical School, Sydney Medical School, Edward Ford Building A27, The University of Sydney, NSW 2006, Australia; 15 Department of Medical Oncology, Westmead Hospital, Darcy Road, Westmead, NSW 2145, Australia; 16 Breast Cancer Institute, Westmead Hospital, Darcy Road, Westmead, NSW 2145, Australia; 17 Western Clinical School, Sydney Medical School, Edward Ford Building A27, The University of Sydney, NSW 2006, Australia; 18 Australian National University and Department of Radiation Oncology, Canberra Hospital, Yamba Drive, Garran, Australian Capital Territory 2605, Australia

## Abstract

**Introduction:**

RAD21 is a component of the cohesin complex, which is essential for chromosome segregation and error-free DNA repair. We assessed its prognostic and predictive power in a cohort of *in situ *and invasive breast cancers, and its effect on chemosensitivity *in vitro*.

**Methods:**

RAD21 immunohistochemistry was performed on 345 invasive and 60 pure *in situ *carcinomas. Integrated genomic and transcriptomic analyses were performed on a further 48 grade 3 invasive cancers. Chemosensitivity was assessed in breast cancer cell lines with an engineered spectrum of RAD21 expression.

**Results:**

RAD21 expression correlated with early relapse in all patients (hazard ratio (HR) 1.74, 95% confidence interval (CI) 1.06 to 2.86, *P *= 0.029). This was due to the effect of grade 3 tumors (but not grade 1 or 2) in which RAD21 expression correlated with early relapse in luminal (*P *= 0.040), basal (*P *= 0.018) and HER2 (*P *= 0.039) groups. In patients treated with chemotherapy, RAD21 expression was associated with shorter overall survival (*P *= 0.020). *RAD21 *mRNA expression correlated with DNA copy number, with amplification present in 32% (7/22) of luminal, 31% (4/13) of basal and 22% (2/9) of HER2 grade 3 cancers. Variations in *RAD21 *mRNA expression in the clinical samples were reflected in the gene expression data from 36 breast cancer cell lines. Knockdown of *RAD21 *in the MDA-MB-231 breast cancer cell line significantly enhanced sensitivity to cyclophosphamide, 5-fluorouracil and etoposide. The findings for the former two drugs recapitulated the clinical findings.

**Conclusions:**

RAD21 expression confers poor prognosis and resistance to chemotherapy in high grade luminal, basal and HER2 breast cancers. RAD21 may be a novel therapeutic target.

## Introduction

Cohesin is a multi-protein complex that is highly conserved from yeast to humans. Its primary role is to adhere sister chromatids in close apposition, a mechanism termed 'sister chromatid cohesion' (SCC). SCC is fundamental to several key cellular processes, including chromosome segregation during mitosis and meiosis, error-free homologous recombinational repair (HRR) of DNA double strand breaks and the regulation of gene transcription [1-7]. The core cohesin complex consists of four proteins, RAD21 (also known as SCC1 or MCD1), SMC1, SMC3 and SCC3 [8].

RAD21 is a central component of the cohesin complex, both structurally and functionally [8]. Aberrant *RAD21 *expression has been reported in multiple cancers and cancer cell lines [9-12]. In a mega-scale microarray analysis of multiple cancers, *RAD21 *was identified as one of 69 signature genes in undifferentiated cancers that had aggressive *in vitro *or clinical courses and poor patient outcomes [9]. Further, an intronal single nucleotide polymorphism (SNP) in the *RAD21 *gene is strongly associated with increased breast cancer risk [10]. Although these reports support the notion that the abnormal activity of RAD21 may be an important feature of human breast cancer, there are no data available from clinical breast cancer samples.

We therefore, evaluated RAD21 expression in a cohort of well-characterised human *in situ *and invasive breast cancers to 1) assess the correlation between RAD21 expression, and conventional and molecular clinicopathological parameters and patient prognostic data; and 2) determine whether aberrant RAD21 expression might predict therapeutic outcomes.

## Materials and methods

### Patient clinicopathological variables

The flow of patients through the study according to the reporting recommendations for tumor marker prognostic studies (REMARK) criteria [13] is listed in Additional file 1. Four hundred and nine invasive cancers were obtained from the Garvan Institute (292 cases with survival and treatment data) and the Peter MacCallum Cancer Centre (117 cases without survival data). Sixty-four cases were excluded due to lack of tissue available for tissue microarray (TMA) construction or absence of tumor on the array. The final cohort of invasive cancers was 345 cases (251 cases with survival data). For ductal carcinoma *in situ *(DCIS), 60 cases of pure DCIS were obtained from the John Radcliffe Hospital, UK, and were assessed on TMAs. This study has Ethics Committee approvals (numbers 00/81, 03/90, 09/36, JRC02.216, HREC SVH H94/080 and HREC SVH 06336 H00036). Patient median age was 54 years (range 24 to 87 years). Forty-one percent of patients received adjuvant chemotherapy with cyclophosphamide/methotrexate/5-fluorouracil (CMF), or doxorubicin (adriamycin)/cyclophosphamide (AC). Fifty-two percent received adjuvant endocrine therapy with tamoxifen. Patient median follow-up was 58.1 months. During this time, 100 patients developed recurrence (24.4%) and 86 deaths (21.0%) were considered breast-cancer related.

### Immunohistochemistry

TMAs were constructed from 1 mm diameter (invasive cancers) or 2 mm cores (DCIS). Sections of 4 μm thickness were used for immunostaining using the EnVision™kit (DAKO, Glostrup, Denmark) following the manufacturer's instructions. Briefly, de-paraffinized and rehydrated tissue sections were treated for antigen retrieval in 10 mM Tris-HCl (pH 9.0) buffer containing 1 mM EDTA for three minutes at 125°C in a pressure cooker (Biocare Decloker, Concord, CA, USA). Sections were then treated with 3% H_2_O_2 _for five minutes to remove endogenous peroxides, washed and incubated with a rabbit polyclonal anti-RAD21 antibody (1:200) (Abcam, Cambridge, UK), with anti-rabbit IgG as negative control, for two hours at room temperature. Horseradish peroxidase (HRP)-conjugated secondary antibody, the signal was detected using DAB (3', 3'-diaminobenzidine) substrate. Sections were counter-stained with hematoxylin to visualize nuclei. Validation of the anti-RAD21 antibody was performed using small interference RNA (siRNA) knockdown of the human *RAD21 *gene in MCF10A cells on cell blocks (Additional file 2).

Nuclear RAD21 expression was assessed for intensity (0 = no staining, 1 = weak, 2 = moderate, 3 = strong) and the percentage of positive cells (0 = 0%, 1 ≤10%, 2 = 10% to 50%, 3 = 51% to 80%, 4 ≥80% positive cells) as defined previously [14]. The scores for intensity and percentage were added and a cut-off of 7 was used to define two approximately equal size groups of patients for subsequent statistical analyses.

ER, HER2, EGFR and CK5/6 staining were used to classify tumors into four intrinsic subgroups: the basal group (ER negative, HER2 negative, CK5/6 and/or EGFR positive), luminal group (ER positive, HER2 negative), HER2 group (HER2 positive) and the negative (null) group (ER, HER2, CK5/6 and EGFR negative) [15].

#### Cell lines

Cell lines (MCF7, MCF10A, MDA-MB-231, MDA-MB-468, SK-BR-3, T47 D and ZR75-1) were obtained from the American Type Culture Collection (ATCC, Rockville, MD, USA). MCF10A was grown and maintained in a 1:1 mixture of Dulbecco's modified Eagle medium (DMEM) F-12 supplemented with 5% horse serum, 20 ng of epidermal growth factor (EGF) per ml, 10 μg/ml of insulin, and 0.5 μg/ml of hydrocortisone. MDA-MB-468, SK-BR-3 and ZR75-1 were grown in MEM with 10% fetal bovine serum (FBS) and 1% Pen/Strep. Other cell lines were grown in RPMI-1640 with 10% FBS and 1% Pen/Strep. SVCT was obtained from European Collection of Cell Cultures (ECACC; Salisbury, Wilts, UK) and grown in DMEM with 10% FBS, 5 μg/ml hydrocortisone, and 10 μg/ml insulin.

#### Quantitative real time PCR and Semi-quantitative Western blot analysis

Exponentially growing cells were harvested and total RNA was extracted using RNeasy kits (Qiagen, Valencia, CA, USA). DNA was removed by on-column treatment with RNase-free DNase. One microgram of total RNA was used for cDNA synthesis using a Superscript III kit (Invitrogen, Carlsbad, CA, USA). cDNA was used for quantitative real time polymerase chain reaction (qRT-PCR) using primers (5'-AATTTGGCTAGCGGCCCAT-3' and 5'-TGTCCGTAATGCCATTTTCACC-3') which span exon 10 and exon 11 of the human *RAD21 *gene. Phosphoglycerate kinase (PGK) was used as a housekeeping gene and the relative expression of *RAD21 *gene was determined from three independent experiments using the DeltaDelta CT method [16].

Total protein extraction and Western blots were carried out essentially as described previously [17]. For Western blot analysis, blots were incubated with a rabbit polyclonal anti-RAD21 antibody (Abcam, Cambridge, UK) followed by a fluorescence-conjugated secondary antibody, Alexa 680 anti-rabbit (Invitrogen, Carlsbad, CA, USA). The signal intensity was measured using the Li-Cor Odyssey system. Membranes were then probed with a mouse monoclonal anti-pan actin antibody (Cell Signaling Technology, Danvers, MA, USA) followed by an IRDye800-conjugated anti-mouse antibody (Rockland, Gilbertsville, PA, USA). The relative level of protein expression was determined by normalizing to the pan-actin loading control from a minimum of three independent experiments.

### Array CGH and gene expression analysis

Array comparative genomic hybridization (CGH) and microarray-based expression profiling were obtained from analysis of 48 microdissected grade 3 invasive ductal carcinomas as described [18]. Tumor subtypes were determined as described above [15].

### Gene expression data mining

Gene expression profiles of 38 breast cancer cell lines were obtained by mining a microarray dataset described by Hollestelle *et al*. (2009) [19] at GEO (accession number (GEO:GSE16795)) [20]. Raw intensity values of all samples were normalized by RMA normalization (Robust Multichip Analysis) (background correction and normalization) using Partek version 6.4 (Partek Inc., St. Louis, MO). The normalized data file was transposed, back transformed to normal intensity values and imported into OmniViz version 6.0.1 (BioWisdom Ltd., Cambridge, UK) for further analysis. For each probe set, the geometric mean of the hybridization intensity of all samples was calculated. The level of expression of each probe set was determined relative to this geometric mean and ^2^log-transformed. The geometric mean of the hybridization signal of all samples was used to ascribe equal weight to gene expression levels with similar relative distances to the geometric mean. Next a query-by-example numerical query was performed in OmniViz to find records most closely related to *RAD21*. The top 25 genes that correlated the best with *RAD21 *were visualized using a treescape view.

### Generation of stable RAD21 knockdown cell lines and clonogenic survival assays

A single cell clone was derived from the human breast cancer cell line, MDA-MB-231. This cell line is of the basal subtype. Two different short-hairpin RNA (shRNA) shRNAmir constructs, shRNA 57223 and shRNA 57224, and shRNAmir vector control (Open Biosystems, Huntsville, AL, USA) were introduced into cells using Lipofectamine 2000 (Invitrogen, Carlsbad, CA, USA) and clones were selected following a two-week culture in 2 μg/ml puromycin. Work involving recombinant DNA was conducted in approved Physical Containment level 2 (PC2) facilities. Western blot analysis and quantitative real-time PCR were used to verify the level of RAD21 protein and mRNA.

Clonogenic survival was performed as described [21]. Exponentially growing cells were seeded in triplicate plates, allowed to adhere for four to six hours. Following 24-hour incubation with individual drugs at graded concentrations, cells were cultured for two weeks. Surviving colonies defined as containing more than 50 cells were counted. Three independent experiments were performed for each treatment. Survival curves were generated using the linear quadratic model, GraphPad Prism version 5.01 for Windows, GraphPad Software, San Diego, CA, USA.

### Statistical analysis

Correlations were examined using the one-way ANOVA, Students *t*-test, or chi-square test where appropriate. Kaplan-Meier survival curves were calculated using tumor recurrence (relapse free survival) and breast cancer-related death (overall survival) as the endpoints and compared using a log rank test. Binary logistic regression was used for multivariate analyses and the Cox proportional hazard regression model was used to identify independent prognostic factors for disease-free and overall survival. Analyses were performed with SPSS 16.0 (SPSS Inc., IL, USA). A two-tailed *P-*value test was used in all analyses and a *P-*value of less than 0.05 was considered statistically significant.

## Results

### RAD21 protein expression in *in situ *and invasive breast cancer

**Figure 1:**
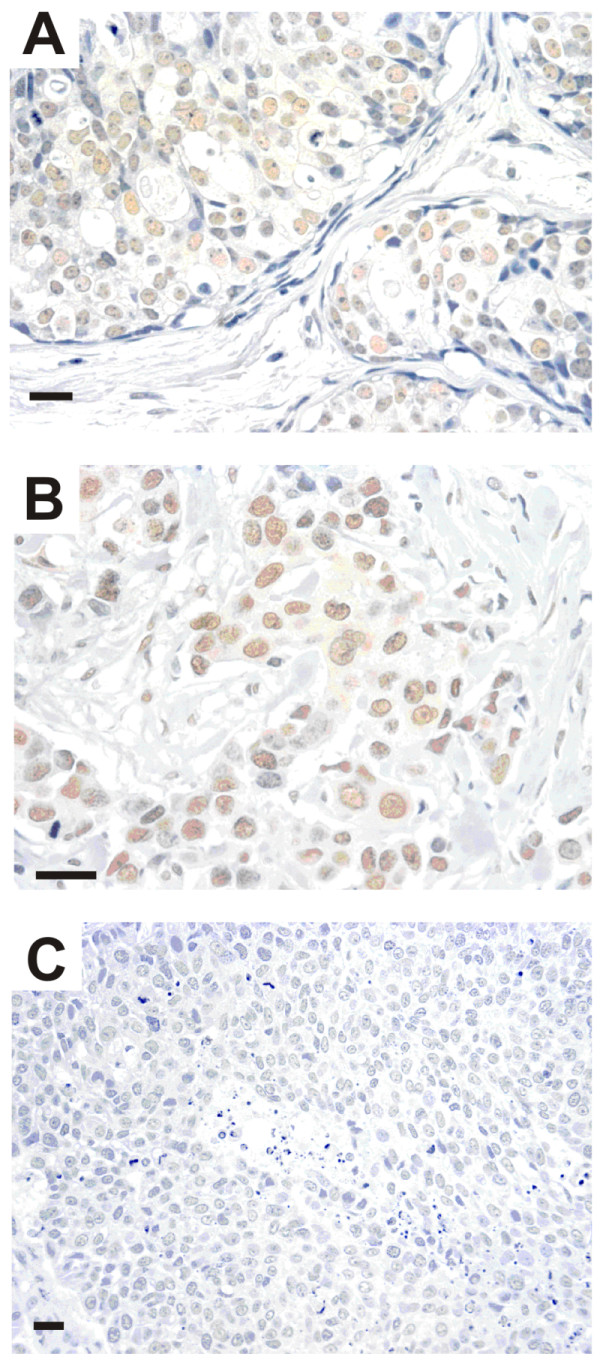
**RAD21 immunohistochemistry in DCIS and invasive carcinoma**. **A**, Strong nuclear RAD21 staining in DCIS. Scale bar = 20 μm. **B**, Strong nuclear RAD21 staining in an invasive carcinoma, luminal type. Scale bar = 20 μm. **C**, Absence of nuclear staining in an invasive carcinoma, basal type. Scale bar = 20 μm.

Expression of RAD21 in DCIS ranged from negative (2/60 cases, 3%) to heterogenous staining (30/60 cases, 50%) and homogenous strong staining (28/60 cases, 47%) (Figure 1A). For invasive cancers, RAD21 staining patterns were similar to DCIS, ranging from heterogeneous (50/95 cases, 53%) and homogeneous strong staining (42/95 cases, 44%) to negative (3/95 cases, 3%) (Figure  1B, C). No staining or minimal weak staining was present in the cytoplasm in all cases. Expression of RAD21 in invasive cancers was significantly lower (104/343, 30%) than in *in situ *cancers (28/60, 47%) (*P *= 0.001).

### Association between RAD21 expression and clinicopathological characteristics in DCIS

RAD21 expression did not significantly associate with nuclear grade (*P *= 0.428) (Table 1) or the intrinsic phenotypes in DCIS (*P *= 0.471) (Table 2). There was no correlation between RAD21 expression in DCIS and risk of relapse (*P *= 0.834).

**Table 1:** RAD21 expression by DCIS grade (*P *= 0.428)

*Grade*	*Negative* *n (%)*	*Positive* *n (%)*	*Total* *n (%)*
***Low***	*3 (50.0%)*	*3 (50.0%)*	*6 (100.0%)*
***Intermediate***	*9 (60.0%)*	*6 (40.0%)*	*15 (100.0%)*
***High***	*8 (38.1%)*	*13 (61.9%)*	*21 (100.0%)*

**Total**	**20 (47.6%)**	**22 (52.4%)**	**42 (100.0%)**

DCIS, ductal carcinoma *in situ*.

**Table 2:** RAD21 expression in DCIS subtypes (*P *= 0.471)

*Subtype*	*Negative* *n (%)*	*Positive* *n (%)*	*Total* *n (%)*
***Luminal***	*21 (55.3%)*	*17 (44.7%)*	*38 (100%)*
***Basal***	*0 (.0%)*	*1 (100.0%)*	*1 (100%)*
***HER2***	*7 (43.8%)*	*9 (56.2%)*	*16 (100%)*
***Null***	*3 (75.0%)*	*1 (25.0%)*	*4 (100%)*

**Total**	**31 (52.5%)**	**28 (47.5%)**	**59 (100.0%)**

DCIS, ductal carcinoma *in situ*.

### Association between RAD21 expression and clinicopathological characteristics and intrinsic subtypes in invasive cancer

RAD21 expression correlated with larger tumor size (*P *= 0.012) and lymph node involvement (*P *< 0.001), but not with tumor grade (*P *= 0.328), age (*P *= 0.815), HER2 status (*P *= 0.564) or ER status (*P *= 0.054) (Table 3). Positive RAD21 expression was seen in 37% (75/201) luminal, 24% (10/42) basal, 22% (9/49) HER2 and 18% (5/28) null, cancers. When compared to luminal cancers, null type cancers, but not basal and HER2 cancers, were significantly more likely to be RAD21 negative (*P *= 0.043) (Table 4).

**Table 3:** RAD21 expression in invasive carcinoma by clinicopathological parameters

	*negative* *n (%)*	*positive* *n (%)*	*Total* *n (%)*	*P value*
**Grade**				***P = 0.328***
***1***	*42 (17.6%)*	*16 (15.4%)*	*58 (16.9%)*	
***2***	*96 (40.2%)*	*35 (33.7%)*	*131 (38.2%)*	
***3***	*101 (42.3%)*	*53 (51.0%)*	*154 (44.9%)*	
**Size**				***P = 0.012***
***<20 mm***	*147 (61.8%)*	*50 (47.2)*	*197 (57.3%)*	
***>20 mm***	*91 (38.2%)*	*56 (52.8%)*	147 (42.7)	
**Lymph node**				***P < 0.001***
***Negative***	*148 (63.5%)*	*43 (42.2%)*	*191 (57.0%)*	
***Positive***	*85 (36.5%)*	*59 (57.8%)*	144 (43.0%)	
**Age**				***P = 0.815***
***<50***	*84 (35.1%)*	*39 (36.4%)*	*123 (35.5%)*	
***>50***	*155 (64.9)*	*68 (63.6%)*	223 (64.5%)	
**ER**				***P = 0.054***
***Negative***	*63 (34.4%)*	*14 (21.5%)*	*77 (31/0%)*	
***Positive***	*120 (65.6%)*	*51 (78.5%)*	171 (69.0%)	
***HER2***				***P = 0.564***
***Negative***	*142 (79.8%)*	*54 (83.1%)*	*196 (80.7%)*	
***Positive***	*36 (20.2%)*	*11 (16.9%)*	47 (19.3%)	
Chemotherapy*				***P = 0.032***
***No***	*117 (62.95)*	*31 (47.7%)*	*148 (59.0%)*	
***Yes***	*69 (37.1%)*	*34 (52.3%)*	*103 (41.0%)*	
**Endocrine therapy**				***P = 0.034***
***No***	*97 (52.2%)*	*24 (36.9%)*	*121 (48.2%)*	
***Yes***	*89 (47.8%)*	*41 (63.1%)*	130 (51.8%)	

* Chemotherapy was given either as cylcophosphamide/methotrexate/fluorouracil (CMF), or doxorubicin (adriamycin)/cyclophosphamide (AC).

ER, oestrogen receptor.

**Table 4:** RAD21 expression in breast cancers by intrinsic subtype

Subtype	*negative* *n (%)*	*positive* *n (%)*	*Total* *n (%)*	*P-value* *Relative to luminal*
**Luminal**	*126 (63%)*	*75 (37%)*	*201 (100%)*	**-**
**Basal-like**	*32 (76%)*	*10 (24%)*	*42 (100%)*	**0.095**
**HER2**	*38 (78%)*	*11 (22%)*	*49 (100%)*	**0.050**
**Null**	*23 (82%)*	*5 (18%)*	*28 (100%)*	**0.043**

**Total**	**239 (70%)**	**104 (30%)**	**320 (100%)**	

### RAD21 expression and its correlation with relapse-free survival

**Figure 2:**
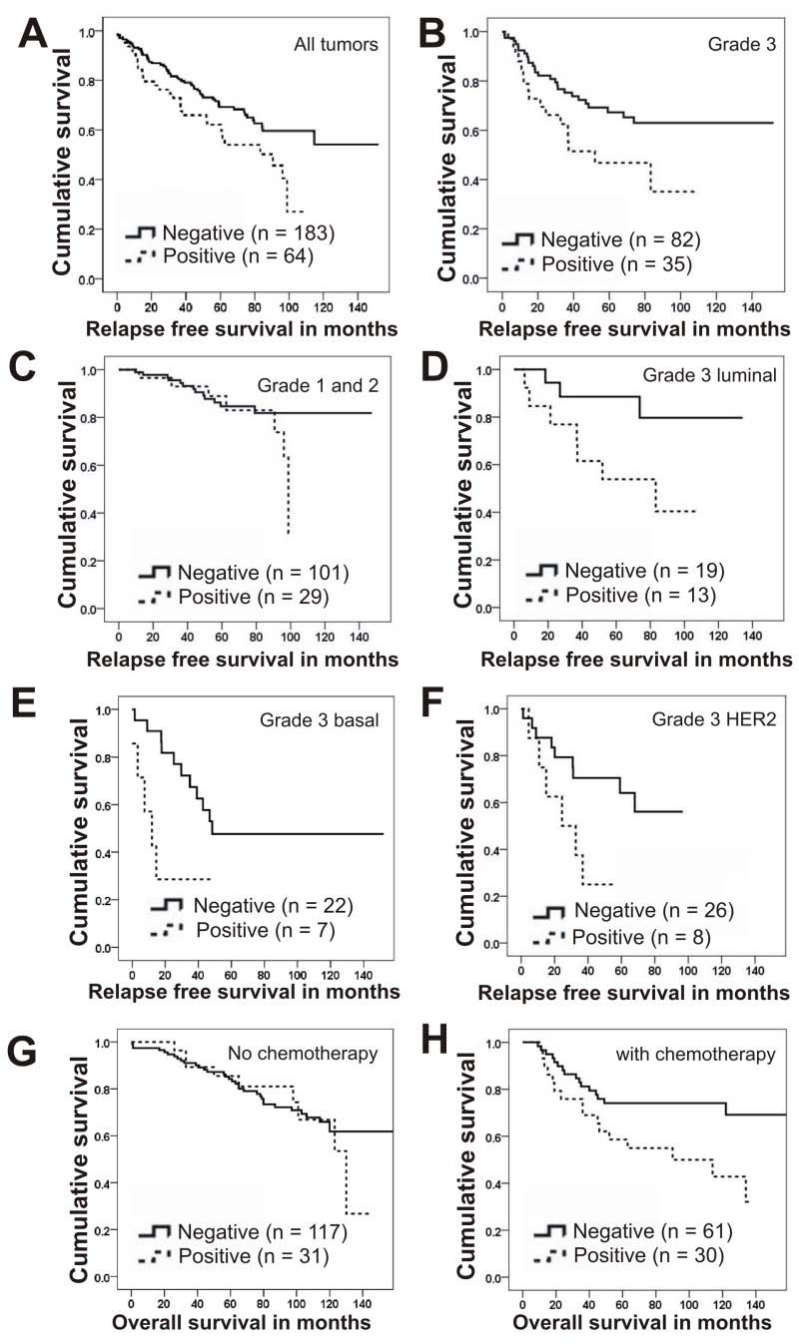
**Kaplan-Meier curves stratified by nuclear RAD21 expression, relapse-free survival (A-F) and overall survival (G-H)**. Relapse free survival: A, in all tumors, (*P *= 0.009) (*n *= 247); B, grade 3 cancers (*P *= 0.023) (*n *= 117); C, grade 1 and 2 cancers (*P *= 0.342) (*n *= 130); D, grade 3 luminal cancers (*P *= 0.040) (*n *= 32); E, grade 3 basal cancers (*P *= 0.018) (*n *= 29); F, grade 3 HER2 cancers (*n *= 34); Overall survival: G, without chemotherapy (*P *= 0.779) (*n *= 148). H, treated with chemotherapy (*P *= 0.020) (*n *= 91).

There was a significant correlation between positive RAD21 expression and shorter relapse-free survival (RFS) (*P *= 0.009) (Figure 2A). The association with early relapse was confirmed on multivariate analysis (*P *= 0.029, HR = 1.74, 95% CI 1.06 to 2.86) (Table 5). Subset analysis revealed RAD21 expression correlated with relapse in grade 3 (*P *= 0.023) (Figure 2B) but not in grade 1 or 2 tumors (*P *= 0.342) (Figure 2C). Further analysis of grade 3 tumors according to subtype showed a significant correlation between RAD21 expression and shorter RFS in the grade 3 luminal (*P *= 0.040) (Figure 2D), grade 3 basal (*P *= 0.018) (Figure 2E) and grade 3 HER2 cancers (*P *= 0.039) (Figure 2F), but not null type cancers (*P *= 0.247).

**Table 5:** Multivariate analysis, Cox regression model of relapse-free survival in all breast cancers

	*P value*	*Hazard Ratio*	*95% CI for hazard ratio*
**RAD21**	0.029	1.74	1.06 to 2.86
**Grade**	0.018	1.63	1.09 to 2.44
**Size > 20 mm**	0.656	1.11	0.71 to 1.73
**Lymph node status**	0.023	1.69	1.07 to 2.65
**ER**	0.003	0.46	0.28 to 0.77
**Age > 50**	0.325	1.28	0.781 to 2.11

CI, confidence interval; ER, oestrogen receptor.

### RAD21 expression and its correlation with overall survival in patients treated with chemotherapy

Among patients not treated with chemotherapy, there was no correlation between RAD21 expression and overall survival (*P *= 0.779) (Figure 2G), whereas among patients treated with chemotherapy there was a significantly shorter overall survival in patients whose tumors were positive for RAD21 expression (*P *= 0.020) (Figure 2H). This association is also true for patients with grade 3 tumors (*P *= 0.021). No significant difference in overall survival was seen in patients treated with endocrine therapy, when stratified by RAD21 expression (*P *= 0.231).

### *RAD21 *gene expression correlates with copy number alterations, and *RAD21 *is amplified in a subset of grade 3 luminal, basal and HER2 cancers

In view of the correlation of RAD21 expression with prognosis in grade 3 cancers, we examined *RAD21 *mRNA expression for its association with gene copy number, in an integrated array CGH and transcriptional dataset generated from 48 microdissected grade 3 invasive ductal carcinomas of luminal (*n *= 22), basal-like (*n *= 13) and HER2 (*n *= 13) subtypes [18]. Array CGH and microarray expression profiling showed *RAD21 *mRNA expression correlated with gene copy number in luminal (*P *= 0.003), basal (*P *= 0.0086) and HER2 (*P *= 0.0035) tumors (Pearson correlation, Table 6). *RAD21 *amplification is present in 32% (7/22) of luminal, 31% (4/13) of basal and 22% (2/9) of HER2 subtypes. These proportions were very similar to our immunohistochemistry analysis of a different sample set described above, where 30% of luminal (14/46), 25% of basal (10/40), and 22% of HER2 (9/41) grade 3 cancers showed positive RAD21 expression. Collectively, these data suggest that positive RAD21 expression observed in a subset of grade 3 tumors may be due to gene amplification.

**Table 6:** Correlation of *RAD21 *gene expression with genomic alterations*

*Subtype*	Copy number Pearson correlation *P*-value	Gaina Mann Whitney U test *P*-value	Amplificationb Mann Whitney U test *P*-value
***Luminal (n = 22)***	0.0030	***0.0169***	***0.0465***
***Basal-like (n = 13)***	***0.0086***	***--***	***0.0111***
***HER2 (n = 13)***	***0.0035***	***0.0503***	***0.1025***

* Patients and Methods were as described in Natrajan *et al*. (2009).

^a ^Gain corresponds to approximately three to five copies of the locus as defined using a smoothed Log2 ratio of between 0.08 and 0.45.

^b ^Amplification was defined as having a Log2 ratio > 0.45, corresponding to more than five copies.

CGH, comparative genomic hybridization.

### *RAD21 *expression in breast cancer cell lines

**Figure 3:**
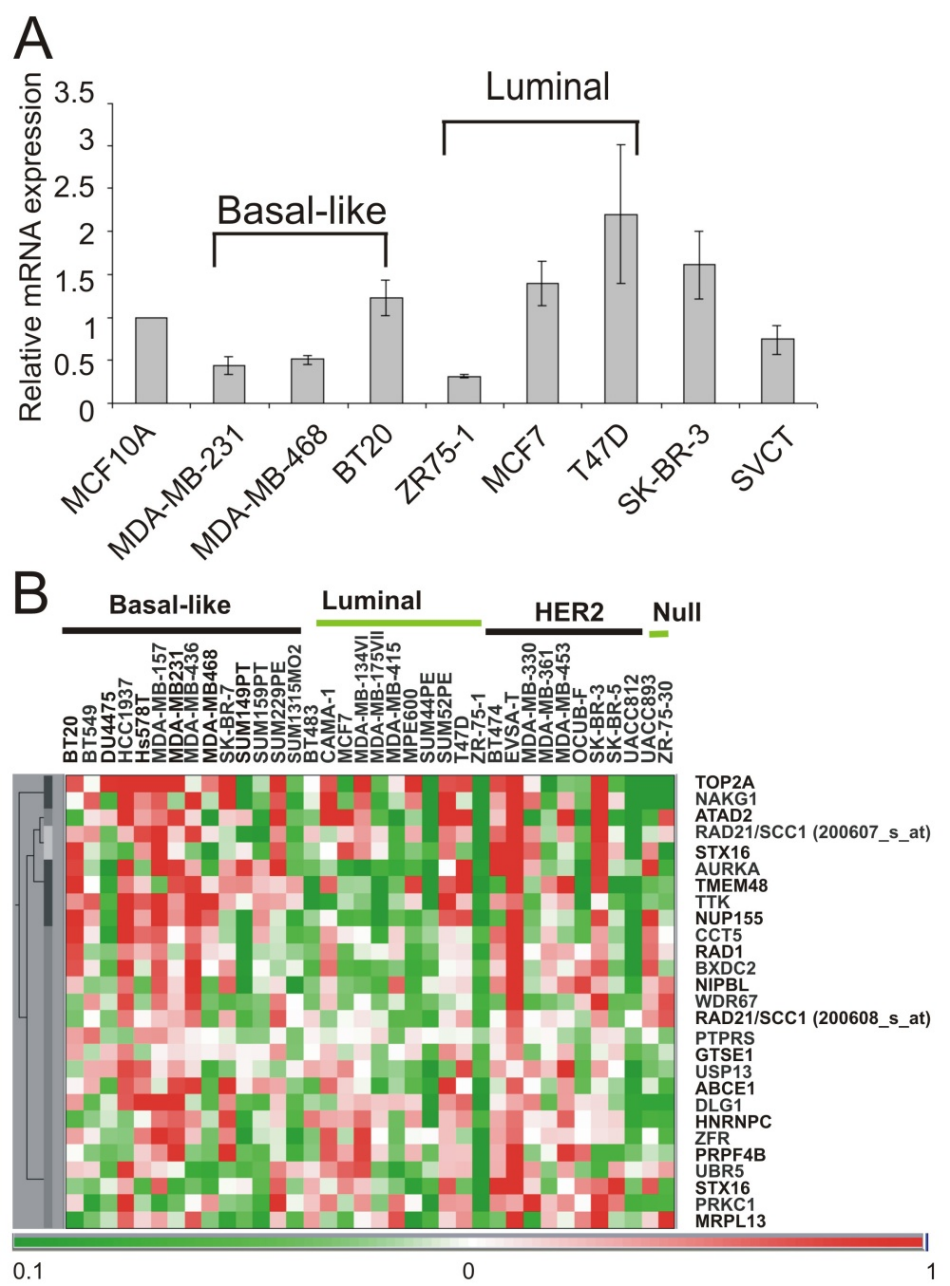
**Expression of *RAD21 *in breast cancer cell lines**. **A**. Quantitative real time RT-PCR of *RAD21 *transcripts in human breast cancer cell lines. The expression level in MCF10A was used as a reference and given an arbitary value of 1. Relative expression of *RAD21 *gene was compared with MCF10A. Error bar = standard error mean (SEM). **B**. OmniViz Treescape showing the hierarchical clustering of the top 25 genes that correlated best with the two *RAD21 *gene probes (200607_s_at and 200608_s_at). Gene expression levels: red, up-regulation compared with the geometric mean; green, down-regulation compared with the geometric mean. The color intensity correlates with the degree of change. Raw dataset (GEO:GSE16795) was sourced from GEO [20].

Variations in RAD21 protein expression in clinical samples were reflected by gene expression analysis using qRT-PCR of a panel of breast cancer cell lines (Figure 3A), and by microarray profiling of 36 breast cancer cell lines derived from Hollestelle *et al*. [19] (Figure 3B). Further analysis revealed that *TOP2A *which encodes topoisomerase II (a protein also required for sister chromatid separation) and *NIPBL *(encoding a cohesin loading protein) are among top 25 genes positively correlated with *RAD21 *expression (Figure 3B).

### Knockdown of *RAD21 *gene expression with short-hairpin RNA in a basal-like breast cancer cell line, MDA-MB-231, results in its enhanced sensitivity to chemotherapeutic drugs

**Figure 4:**
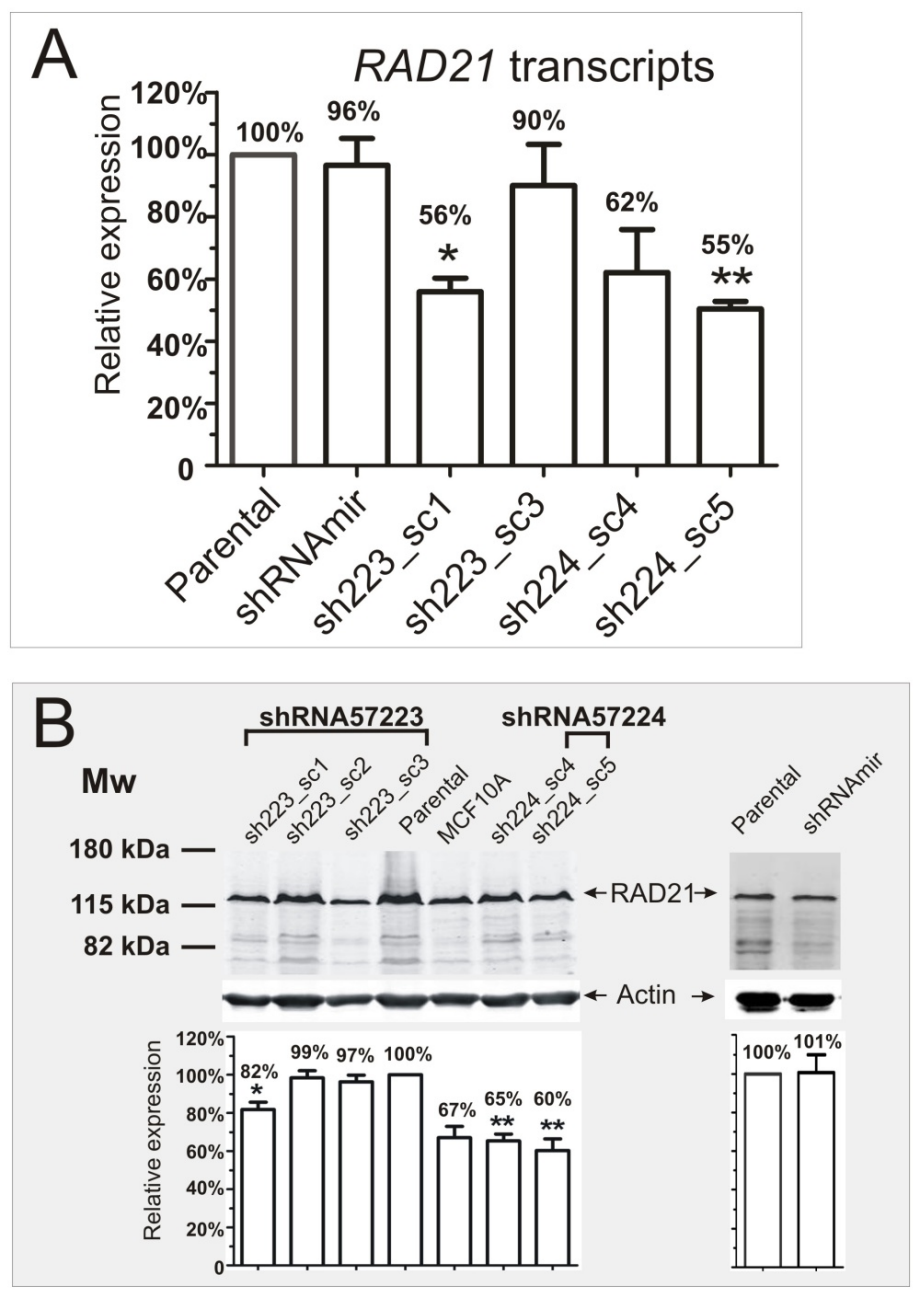
**Validation of *RAD21 *expression in MDA-MB-231 cells carrying shRAD21 knockdown constructs**. **A**. Quantitative real time PCR analysis of *RAD21 *expression in clones stably expressing two different shRAD21 constructs and shRNAmir vector, relative to the parental cell line. **B**. Western blot analysis of RAD21 protein level. RAD21 expression in five independently derived clonal cell lines with two different stable *RAD21 *knockdown constructs was compared to the parental cell line, cells transfected with shRNAmir vector and an immortalized human breast epithelial cell line MCF10A. Pan-actin was used as loading control. A reduction in the levels of RAD21 protein in three stable clones (sh223_sc1, sh224_sc4 and sh224_sc5) was verified by semi-quantitative Western blot analysis (bottom panel). The levels of RAD21 protein were normalized to the pan-actin loading control and expressed as the percentage of the parental line where the RAD21 expression was given an arbitrary value of 100%. The values represent the mean of four independent experiments except for shRNAmir vector and MCF10A where three independent experiments were performed. Clones with a significant reduction in either *RAD21 *mRNA or protein levels were marked by asterisks (* *P *< 0.05; ** *P *< 0.005, Student *t*-test). Error bar = SEM.

To test the functional significance of our cancer therapy results that RAD21 expression affects sensitivity to chemotherapeutic drug response, we used a small hairpin shRNA-mediated gene-silencing approach to knockdown the *RAD21 *gene in MDA-MB-231 breast cancer cell line. Of several independent cell clones generated using two different shRNA constructs, three clones exhibited a reduction in both *RAD21 *transcripts and protein (Figure 4). The relative levels of *RAD21 *mRNA in four stable clones, as determined by qRT-PCR analysis, were 56 ± 4% for sh223_sc1 (*P *= 0.010), 90 ± 13% for sh223_sc3 (*P *= 0.531), 62 ± 13% for sh224_sc4 (*P *= 0.111) and 55 ± 2% for sh224_sc5 (*P *= 0.002), relative to the parental cell line (Figure 4A). No apparent reduction in *RAD21 *mRNA was detected in the control clone transfected with shRNAmir vector (96% ± 8%, *P *= 0.726) (Figure 4A). Further examination of the corresponding RAD21 protein by semi-quantitative Western blot analysis revealed a statistically significant reduction in the levels of RAD21 protein in the three clones (sh223_sc1, sh224_sc4 and sh224_sc5), which exhibited *RAD21 *mRNA reduction (Figure  4A, B). The relative levels of RAD21 protein were 82% ± 4% for sh223_sc1 (*P *= 0.017), 65 ± 4% for sh224_sc4 (*P *= 0.002) and 60 ± 6% for sh224_sc5 (*P *= 0.007), relative to the parental cell line (Figure 4B). The RAD21 protein levels in the sh224_sc4 and sh224_sc5 clones are comparable to that in an immortalized human mammary epithelial cell line, MCF10A (Figure 4B). No apparent reduction in RAD21 protein was detected in sh223_sc2 (99% ± 4%, *P *= 0.700), sh223_sc3 (97 ± 4%, *P *= 0.367) and shRNAmir vector (101 ± 9%, *P *= 0.947), compared to the parental line (Figure 4B). Thus, reduction of both *RAD21 *mRNA and protein levels was confirmed in three stable shRAD21 knockdown clones.

**Figure 5:**
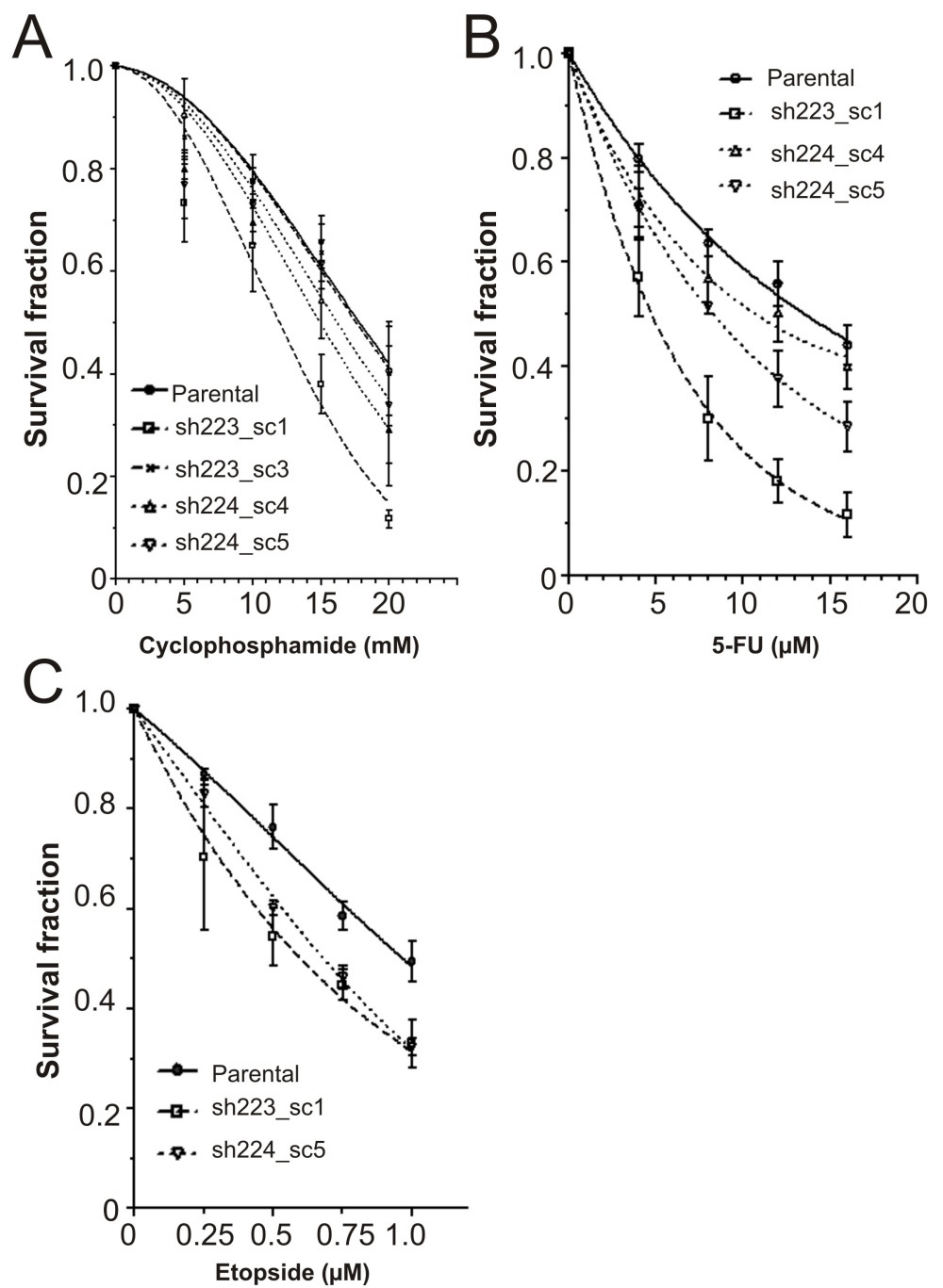
**Effect of shRNA-mediated *RAD21 *knockdown on cellular sensitivity to anti-cancer drugs**. Independent cell clones with stably reduced *RAD21 *expression were derived from the breast cancer cell line, MDA-MB-231. Relative levels of *RAD21 *gene expression when compared to the parental line by qRT-PCR and semi-quantitative Western blot analysis were shown in Figure 4. Error bar = SEM. Clonogenic survival following treatment with: **A**. cyclophosphamide: sh223_sc1 (*P *= 0.0316), sh223_sc3 (*P *= 0.175), sh224_sc4 (*P *= 0.0187) and sh224_sc5 (*P *= 0.0563); **B**. 5-FU: sh223_sc1 (*P *= 0.020), sh224_sc4 (*P *= 0.0257) and sh224_sc5 (*P *= 0.0242); and **C**. etoposide: sh223_sc1 (*P *= 0.020) and sh224_sc5 (*P *= 0.042).

We next tested the response of shRAD21 clones to two breast cancer chemotherapeutic drugs, cyclophosphamide and 5-fluorouracil (5-FU). All three *RAD21 *knockdown clones, sh223_sc1, sh224_sc4 and sh224_sc5, showed increased sensitivity to the drug following treatment with cyclophosphamide, in a manner that directly correlates with the level of RAD21 expression (Figure 5A). In contrast, such enhanced sensitivity was not observed in the clone sh223_sc3 which did not show a reduction in either *RAD21 *mRNA or protein level (Figure 4). We noted that the sh223_sc1 clone exhibited a more reduced clonogenic survival compared to the other clones, sh224_sc4 and sh224_sc5. This may be due to the difference in targeting sequences between the sh223 and sh224 shRNAs. Similarly, treatment of three shRAD21 clones (sh223_sc1, sh224_sc4 and sh224_sc5) with 5-FU resulted in a significant reduction in the clonogenic survival of all three clones compared to the parental line (Figure 5B). These data recapitulate our findings in patients treated with cyclophosphamide/methotrexate/5-fluorouracil (CMF) or doxorubicin (adriamycin)/cyclophosphamide (AC) (Figure 2H), providing further evidence that RAD21 expression correlates with cellular sensitivity to chemotherapeutic-drugs.

Since our analysis showed that *RAD21 *expression strongly correlates with *TOP2A *expression in a number of breast cancer cell lines (see Figure 3), we assessed the sensitivity of *RAD21 *knockdown clones to etoposide, a topoisomerase II inhibitor and commonly used anti-cancer drug. Etoposide treatment also led to a marked decrease in cell survival in the shRAD21 clones tested, compared to the parental cells (Figure 5C). This result is consistent with an early report of an enhanced etoposide-sensitivity following a siRNA-mediated transient *RAD21 *knockdown in MCF7 breast cancer cell line [10].

## Discussion

This translational study is the first comprehensive analysis of a novel chromosomal DNA repair protein, RAD21 cohesin, in breast cancer. Our analyses provide compelling evidence that RAD21 expression is a novel prognostic marker in breast cancer and is also highly predictive of anti-cancer therapy outcomes. Tumor RAD21 overexpression strongly correlated with amplification of the *RAD21 *gene locus in a significant subset of high grade luminal, basal and HER2 cancers. This suggests that the observed RAD21 overexpression resulted from gene amplification, and provides a plausible explanation for the strong RAD21 prognostic effects observed in these tumors. Our findings in breast cancer with the RAD21 cohesin may also generalize to some other epithelial cancer types: consistent with our data, *RAD21 *at chromosomal locus 8q24 is also commonly amplified in advanced androgen-resistant prostate cancer [11].

Our immunohistochemical analysis showed that RAD21 expression associates with shorter relapse-free survival in patients with high grade breast cancer. Based on known RAD21 functions, the adverse outcome in breast cancer patients with RAD21 expression could be due to an elevated level of homologous recombination (HR) repair activity as a result of RAD21 overexpression. We favor this explanation. Overexpression of other HR proteins (for example, RAD51, BRCA1) has also been shown to be associated with increased resistance to radio- and chemo-therapy [22,23]. Furthermore, should RAD21 expression prove to be a surrogate for HR activity, this may provide a simple, cost-effective and novel way for evaluating HR activity on tissue sections. Alternatively, numerical chromosome (ploidy) alterations as a result of chromosome segregation errors could contribute to the poorer survival outcomes we observed.

RAD21 expression is a prognostic and predictive factor that affects the ultimate outcome in many breast cancer sub-histotypes. Prior to clinical use, these data will require validation, for example, in another (larger) clinical dataset, including assessment in a randomised clinical trial. Once validated, RAD21 expression would have potential translational use in the clinical management of patients. Immunohistochemical evaluation of RAD21 protein on breast cancer specimens could be routinely incorporated into standard pathology reporting to estimate levels of RAD21 that might determine drug response of certain drug regimens.

Furthermore, our survival analysis revealed that in patients receiving chemotherapy, those patients with tumors positive for RAD21 expression showed a significantly shorter overall survival than patients whose tumors were negative for RAD21, highlighting an exciting potential role for RAD21 expression in predicting cancer therapy response. We further verified the relevance of RAD21 expression to the therapeutic response *in vitro*. Stable knockdown of *RAD21 *significantly enhanced, in a graded fashion, cellular sensitivity to 5-FU, cyclophosphamide and etoposide. The first two of these drugs are components of the commonly used FEC (5-FU/epirubicin/cyclophosphamide), CMF (cyclophosphamide/methotrexate/5-fluorouracil) and AC (doxorubicin (adriamycin)/cyclophosphamide) regimens for breast cancer. The repair of DNA adducts caused by 5-FU, cyclophosphamide and etoposide depends on HR [24-26] although this dependency on HR is only partial for the repair of cyclophosphamide-induced DNA interstrand crosslinks [27]. The decrease in cell survival that correlated with levels of RAD21 in breast cancer cells after *RAD21 *knockdown, is, therefore, in keeping with the dependence of breast cancer cells on the HR pathway to repair DNA damage from chemotherapy [10]. Consistent with this proposition, we noted that both topoisomerase II and the RAD21 loading protein NIPBL, showed strong coordinately regulated expression with RAD21 in breast cancer cell lines. RAD21 is recruited, in a manner dependent on the cohesin loading protein NIPBL, to the sites of DNA double strand breaks such as those generated by topoisomerase II, promoting DNA repair in human cells [28]. Further, topoisomerase II also decatenates chromosomes before the condensation needed for RAD21 cohesin-mediated chromosome segregation. Collectively, ours and other data highlight the likely effects of the DNA repair functions of RAD21 in tumor biology, and their potential importance in cancer treatment, although other aberrant functions of RAD21 could theoretically contribute to our observed associations and phenotypes.

In conclusion, the predictive function of RAD21 expression for chemotherapy outcome suggests that RAD21 expression could be used to guide treatment selection. For example, in high grade tumors with enhanced RAD21 expression, consideration could be given to using chemotherapeutic drugs that are inhibitors of HR repair [25]. Furthermore, it could guide the use of alternative strategies, such as drug dosage intensification, other chemotherapy drugs with activity in breast cancer, or chemotherapy in combination with radiotherapy (RT) which is a potent inducer of DNA double stand breaks. Further testing of the sensitivity of *RAD21 *knockdown clones to other chemotherapeutic agents may be of utility. Because of its effect on prognosis and therapeutic outcome, enhanced RAD21 expression may also be a novel therapeutic target. Many of the methodologies used to clinically counteract oncogene expression, (for example, gene therapy, antisense oligonucleotide therapy, specific microRNA expression), could be entertained to reduce RAD21 levels.

## Conclusions

In summary, expression of RAD21 in a significant subset of breast cancers confers poor prognosis in high grade luminal, basal and HER2 breast cancers, and resistance to chemotherapy in breast cancer. RAD21 may be a novel marker of poor prognosis, a predictive factor for systemic therapy outcomes and a new target for breast cancer therapy.

## Abbreviations

5-FU: 5-fluorouracil; AC: doxorubicin (adriamycin)/cyclophosphamide; CGH: comparative genomic hybridization; CMF: cyclophosphamide/methotrexate/5-fluorouracil; DAB: 3': 3'-diaminobenzidine; DCIS: ductal carcinoma *in situ*; DMEM: Dulbecco's modified Eagle medium; EGF: epidermal growth factor; HR: homologous recombination; HRP: horseradish peroxidase; HRR: homologous recombinational repair; PC2: Physical Containment level 2; PGK: phosphoglycerate kinase; qRT-PCR: quantitative real time PCR; RFS: relapse-free survival; RMA: Robust Multichip Analysis; RT: radiotherapy; SCC: sister chromatid cohesion; shRNA: short-hairpin RNA; siRNA: small interference RNA; SNP: single nucleotide polymorphism; TMA: tissue microarray.

## Competing interests

The authors declare that they have no competing interests.

## Authors' contributions

HX contributed to the conception and design of the study, performed experiments, data analysis and interpretation, and drafted and revised the manuscript. MY scored and analyzed immunohistochemistry data, performed the statistical analysis, and drafted and revised the manuscript. JP participated in antibody validation, immuno-staining and the generation of RAD21 knockdown cells. RN contributed to the collection, analysis and interpretation of CGH and microarray data, and revised the manuscript. YY participated in immuno-staining, characterization and survival assays of RAD21 knockdown cells. SS and PS carried out the analysis and interpretation of microarray data on cell lines, and revised the manuscript. JMT participated in the gene expression analysis, and revised the manuscript. SV performed qRT-PCR analysis and contributed to data analysis. JSRF contributed to CGH and microarray data analysis and interpretation. EAKM, SAO, CMN and RLS contributed to the study materials, patient data collection, and revised the manuscript. RGR contributed to the conception and design, data analysis and interpretation, and revised the manuscript. MJM contributed to the conception and design of the study, data analysis and interpretation, and drafted and revised the manuscript. SF contributed to the study conception and design, provision of study materials, collection and assembly of data, data analysis and interpretation, and drafted and revised the manuscript. All authors read and approved the final manuscript.

## Supplementary Material

Additional file 1 **Summary of breast cancer patient samples used for the study**. A pdf file containing a table that summarizes breast cancer patient samples used for this study. Click here for file

Additional file 2 **Anti-RAD21 antibody validation**. A pdf file showing the validation of the anti-RAD21 antibody using siRNA-mediated knockdown of the human *RAD21 *gene in MCF10A cells. Click here for file
