# Supplementary material for: Enhanced RAD21 cohesin expression confers poor prognosis and resistance to chemotherapy in high grade luminal, basal and HER2 breast cancers
Source: Breast Cancer Res. 2011 Jan 21;13(1):R9. doi: 10.1186/bcr2814 (PMC3109576; doi:10.1186/bcr2814)
Supplement: Additional file 1 — Summary of breast cancer patient samples used for the study. A pdf file containing a table that summarizes breast cancer patient samples used for this study. [file bcr2814-S1.PDF]

Additional file 1

Additional Table 1. Summary of breast patient samples used for the study.

Tissue type	*Number of cases on TMAs*	*Cases scored for RAD21*	*Cases scored by subtypes*
**DCIS**	***80***	***60***	***59***
**Invasive Carcinoma**			
without prognostic data	*117*	*94*	*93*
with prognostic data	*292*	*251*	*234*
Total	***409***	***345***	***327***
**Total**	**489**	**405**	***361***

DCIS – ductal carcinoma in situ; TMA – tissue microarray
