# Supplementary material for: Enhanced RAD21 cohesin expression confers poor prognosis and resistance to chemotherapy in high grade luminal, basal and HER2 breast cancers
Source: Breast Cancer Res. 2011 Jan 21;13(1):R9. doi: 10.1186/bcr2814 (PMC3109576; doi:10.1186/bcr2814)

Additional file 2

Additional Figure 1. Validation of anti-RAD21 antibody.

(A-B) Validation of the anti-RAD21 antibody in MCF10A cells. The specificity of a rabbit polyclonal anti-RAD21 antibody (Abcam, Cambridge, UK) was tested using small interference RNA (siRNA) to knockdown human *RAD21* gene in MCF10A cells. siRNAs were designed using Biopresi algorithms. Two siRNA were used: siRNA_1 (5'-GGGAGUAGUUCGAAUCUAUTT-3') and siRNA_2 (5'-GGAUGAAUUCCUCAAAGAATT-3'). siRNAs were synthesized by Qiagen (Valencia, CA, USA) and delivered to MCF10A cells using siPORT NeoFX (Ambion, Austin, TX, USA) according to the manufacturer's instructions. **(A)** 48 hours post siRNA transfection, cells were harvested for protein extraction as described.[1] Western blot analysis confirmed a reduced RAD21 protein level in MCF10A cells. Arrow indicates ~120kDa full length RAD21 protein. Two protein bands of lower Mw with weaker intensity were also detected (arrowheads). These bands were presumably cleavage products of RAD21 protein (Weizenegger *et al.*, 2000). A reduction in intensity of ~120kDa full length RAD21 protein, as well as the lower molecular weight bands was observed in cells transfected with siRNA_1. No apparent reduction in RAD21 protein was seen in cells transfected with siRNA_2, presumably this siRNA is less effective in knocking down *RAD21* transcripts. Actin was used as loading control. **(B)** RAD21 immunostaining of MCF10A cells. Cells were fixed in 10% buffered formalin, dehydrated and embedded in paraffin. Sections of 4µm were used for immuno-staining. The great majority of cells in the untransfected control showed a strong nuclear staining (left panel). In cells transfected with siRNA_1, positive staining was present in some nuclei (black arrowheads) while absent in others (red arrows) (right panel).

(C-E) Validation of anti-RAD21 antibody on human breast carcinoma. **(C)** Representative sections of human breast carcinoma immuno-stained for RAD21. Note the strong nuclear staining (brown). Scale bar = 20 µm. **(D)** Representative section stained with anti-rabbit IgG as negative

controls. Scale bar = 20 μm. **(E)** Immunostaining of RAD21 in human breast carcinoma. Note the absence of RAD21 staining on chromosomes of mitotic cells (arrow). Scale bar = 20 μm.

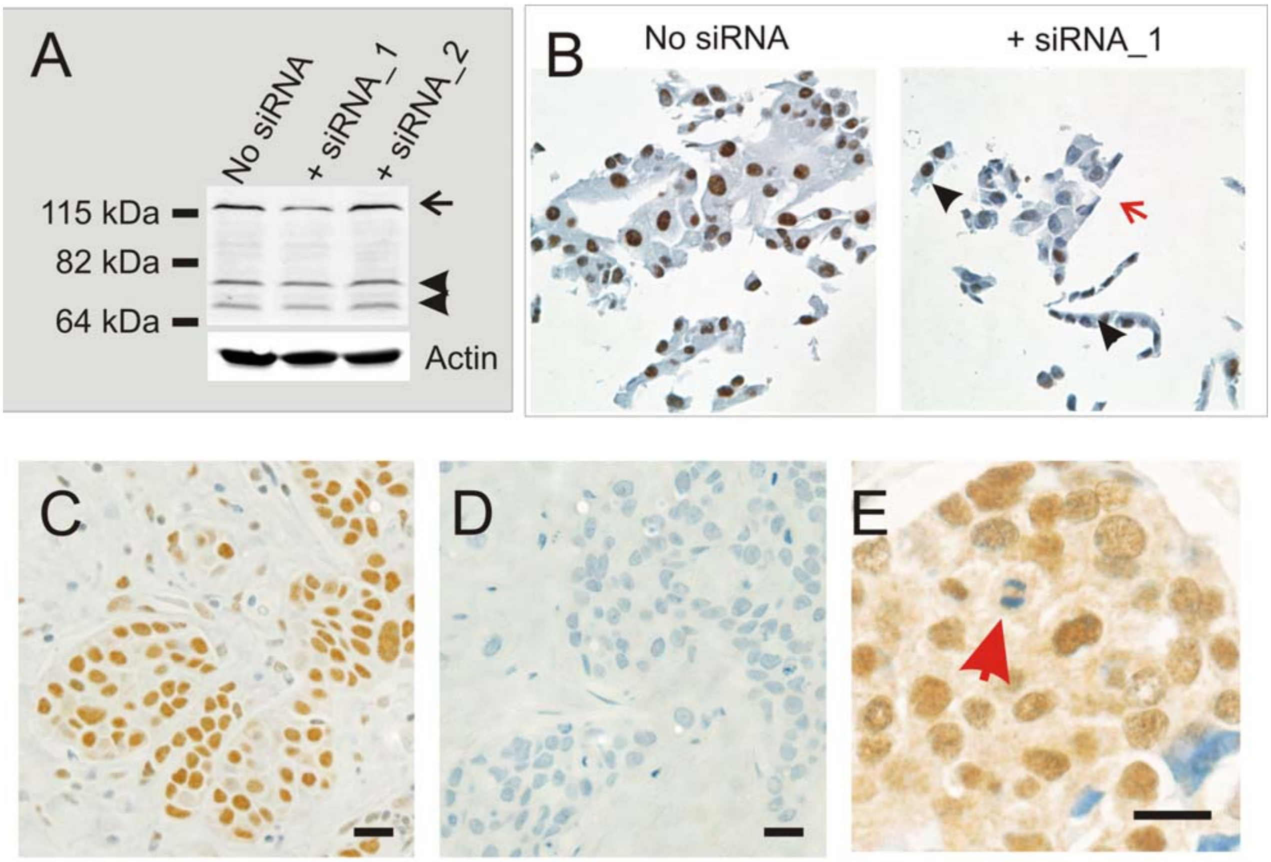

Supplement: Additional file 2 — Anti-RAD21 antibody validation. A pdf file showing the validation of the anti-RAD21 antibody using siRNA-mediated knockdown of the human RAD21 gene in MCF10A cells. [file bcr2814-S2.PDF]
